# Checking Questionable Entry of Personally Identifiable Information Encrypted by One-Way Hash Transformation

**DOI:** 10.2196/medinform.5054

**Published:** 2017-02-17

**Authors:** Xianlai Chen, Yang C Fann, Matthew McAuliffe, David Vismer, Rong Yang

**Affiliations:** ^1^ Institute of Information Security and Big Data Central South University Changsha China; ^2^ Intramural IT and Bioinformatics Program, Division of Intramural National Institute of Neurological Disorders and Stroke National Institutes of Health Bethesda, MD United States; ^3^ Division of Computational Science Center for Information Technology National Institutes of Health Bethesda, MD United States; ^4^ Sapient Government Services Arlington, VA United States; ^5^ 7th Ward Xiangya Hospital Central South University Changsha China

**Keywords:** data accuracy, personally identifiable information, confidentiality, computer security, quality control, medical record linkage, registries, privacy

## Abstract

**Background:**

As one of the several effective solutions for personal privacy protection, a global unique identifier (GUID) is linked with hash codes that are generated from combinations of personally identifiable information (PII) by a one-way hash algorithm. On the GUID server, no PII is permitted to be stored, and only GUID and hash codes are allowed. The quality of PII entry is critical to the GUID system.

**Objective:**

The goal of our study was to explore a method of checking questionable entry of PII in this context without using or sending any portion of PII while registering a subject.

**Methods:**

According to the principle of GUID system, all possible combination patterns of PII fields were analyzed and used to generate hash codes, which were stored on the GUID server. Based on the matching rules of the GUID system, an error-checking algorithm was developed using set theory to check PII entry errors. We selected 200,000 simulated individuals with randomly-planted errors to evaluate the proposed algorithm. These errors were placed in the required PII fields or optional PII fields. The performance of the proposed algorithm was also tested in the registering system of study subjects.

**Results:**

There are 127,700 error-planted subjects, of which 114,464 (89.64%) can still be identified as the previous one and remaining 13,236 (10.36%, 13,236/127,700) are discriminated as new subjects. As expected, 100% of nonidentified subjects had errors within the required PII fields. The possibility that a subject is identified is related to the count and the type of incorrect PII field. For all identified subjects, their errors can be found by the proposed algorithm. The scope of questionable PII fields is also associated with the count and the type of the incorrect PII field. The best situation is to precisely find the exact incorrect PII fields, and the worst situation is to shrink the questionable scope only to a set of 13 PII fields. In the application, the proposed algorithm can give a hint of questionable PII entry and perform as an effective tool.

**Conclusions:**

The GUID system has high error tolerance and may correctly identify and associate a subject even with few PII field errors. Correct data entry, especially required PII fields, is critical to avoiding false splits. In the context of one-way hash transformation, the questionable input of PII may be identified by applying set theory operators based on the hash codes. The count and the type of incorrect PII fields play an important role in identifying a subject and locating questionable PII fields.

## Introduction

### Background

To accelerate biomedical discovery, it is critical for researchers to collaborate, especially to share their study data with each other. After announcing the Big Data Research and Development Initiative to explore how big data could be used to address important problems faced by the government in 2012, Obama’s administration proposed Precision Medicine Initiative [1] in 2015. The latter will seek to collect data from large populations and integrate biomedical research with health care. In general, subject data is collected from multiple sites. There needs to be a link between the data from those different sites on the same subject. Personally identifiable information (PII) is often used to identify and aggregate different types of data (eg, laboratory, imaging, genetic, clinical assessment data) of the same subject collected from multiple sites [2]. Generally PII includes an ID (eg, patient ID, social security number, or national ID), name, birth date, birth place, address, postcode, and so on [3]; however, sharing PII may lead to disclosing privacy of an individual. Therefore, when medical data is shared, privacy protection is a very important task of biomedical research [4,5], especially when PII is a concern [6]. Patient data must be protected before they are transferred [7,8]. In the United States, sharing health information must comply with the Standards for Privacy of Individually Identifiable Health Information and the Common Rule [9,10].

There are various methods to protect a patient’s privacy, including data anonymization [10,11], deidentification [12-14], depersonalization [15], limited dataset [16], and hash transformation [17,18]. Among the unique ID methods of protecting patient privacy, the global unique identifier (GUID) algorithm is an effective solution. It transforms combination patterns of PII fields into hash codes by a one-way hash algorithm. It can be used to identify a participant across sites or studies, without transferring any portion of PII. Multiple PII fields can be gathered and combined in different patterns, facilitating matching even in the face of variations across collection sites. As part of the GUID algorithm, the identifying information undergoes one-way hash before being transferred to the central system, so that PII is never transmitted or stored outside collection sites.

For the GUID system [18] to work properly, PII must be collected with a high degree of accurate entry. If there are many errors in the items captured, none of the hash codes may match and there will be a false split (ie, where the same subject is given 2 different GUIDs). Although several methods, including double data entry, were proposed to improve data entry accuracy, the most effective way is prompting questionable fields during data entry. Therefore, while registering a subject, the client application of the GUID system would ideally check the PII input to allow the user to correct them, if any errors are found. This task must depend on the information stored on the GUID server; however, only the GUID and its related hash codes are stored on the GUID server (ie, no portion of PII is stored on the server). In addition, a GUID is a random code that is not directly generated from PII or hash codes. Hash codes are related to PII, but they have been mapped by a one-way hash algorithm, and it is impossible to reidentify PII fields. Thus, it is problematic to find exact questionable inputs while registering a subject. Fortunately, in the GUID system, there are multiple hash codes, which are transformed from combinations of PII fields and where some of the PII fields are overlapping within different hash codes. Therefore, it is possible to identify and reduce data entry error based on matching hash codes and its corresponding PII fields. Our study will explore it based on set theory.

Before exploring the analysis of questionable data input while registering a subject in the GUID system, it is necessary to review the principle of the system.

### The GUID System

#### PII Fields and Its Combination Patterns

The GUID system [18] uses 17 PII fields for identifying a subject, including 8 required fields and 9 optional fields (Table 1). Generally, they are unique for the subject and do not change in the lifetime of the subject. Each PII field has its associated approximated probability such that 2 different individuals can randomly be identified within the subject population of the system sharing the same value for that field.

Each PII field is programmatically normalized to have only uppercase letters and numbers, no spaces, and no punctuation. For each subject, these PII fields are combined with 5 patterns (Table 2) according to their combined inverse probability that ensures a high degree of subject separation. Each combination pattern is converted into a 64-byte hash code by a one-way hash algorithm. An additional byte is appended to each resulting code to indicate the count of missing PII fields for the hash code. Each combination is sufficient to discriminate confidently subjects. In turn, a random unique GUID code will be generated and associated with that subject. The GUID and its linked hash codes are stored on the GUID server and used for anonymously identifying the subject in a clinical study. Because PII fields are not sent to the GUID server, and therefore are not stored in the server, privacy protection is maintained.

**Table 1:** Personally identifiable information (PII) fields used in global unique identifier (GUID) system.

Type	Name	Meaning
Required	FN	Complete legal given (first) name at birth
	LN	Complete legal family (last) name at birth
	MN	Complete legal additional (middle) name
	SEX	Physical sex at birth (male or female)
	COB	Country of government issued or national ID
	DOB	Day of birth
	MOB	Month of birth
	YOB	Year of Birth
Optional	GIID	Government issued or national ID
	MFN	Mother’s complete legal given (first) name at her birth
	MLN	Mother’s complete legal family (last) name at her birth
	FFN	Father’s complete legal given (first) name at his birth
	FLN	Father’s complete legal family (last) name at his birth
	MDOB	Mother’s day of birth
	MMOB	Mother’s month of birth
	FDOB	Father’s day of birth
	FMOB	Father’s month of birth

**Table 2:** Personally identifiable information (PII) combination patterns for hash cod.

Hash code	Combinations patterns
1	YOB + DOB + SEX + GIID^a^
2	FN + MN + LN + COB + DOB + MOB
3	FN + YOB + MFN^a^+ MLN^a^+ FFN^a^+ FLN^a^
4	FN + LN + COB + SEX + MDOB^a^+ MMOB^a^+ FDOB^a^+ FMOB^a^
5	FN + MN + MOB + MFN^a^+ FFN^a^+ MLN^a^

^a^The field that is optional.

#### Match Rule of Hash Code and Subject in GUID System

**Figure 1:**
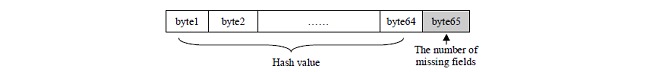
Components of hash code.

As part of the GUID system, each hash code consists of 64-bytes hash value, which is computed from PII combination pattern using a one-way hash algorithm, and 1 additional byte is added to hold the count of missing PII fields in the hash code (Figure 1). So, any error with PII fields used in a combination will result in a failure to match a hash code.

The GUID system has 3 types of hash codes: perfect, good, and bad. For each hash code, 2 parameters are used to determine its type: a lower threshold (L) and an upper threshold (U) (Table 3). A perfect hash code requires that the count of missing PII fields is equal to or less than L. The count of missing PII fields for generating a good hash code is limited to the interval (L,U). If the count of missing PII fields is greater than U, its related hash code will be defined as a bad one. The match between 2 perfect hash codes is called a perfect match, and the match between 2 good hash codes is considered a good match.

**Table 3:** Thresholds of missing fields to determine type of hash code.

Parameters	Hash code 1	Hash code 2	Hash code 3	Hash code 4	Hash code 5
Lower threshold	0	1	1	1	1
Upper threshold	1	2	3	3	3

Once PII is inputted while registering a subject, the system will calculate the count of perfect matches or good matches. In turn, it will determine if there exists a matched subject based on matched hash codes. There are 3 parameters to determine if a subject is matched: threshold for a perfect match (P), threshold for a good match (G), and threshold for a mixed match (X). Two subjects match each other when the count of perfect matches ≥ P, or the count of good matches ≥ G, or the sum of the count of perfect matches and good matches ≥ X. In this system, the thresholds are set to *P*=1, G=2, and X=2. In the context of the above GUID system, correct PII is critical for uniquely identifying a subject. Therefore, before requesting a randomly assigned GUID from the server, checking the input value of the PII fields is essential; however, since hash code is the only information related to PII in the GUID system, a process for checking questionable PII input must depend on the hash codes.

## Methods

### Study Design

Hash codes are generated from the combinations of PII fields in GUID system, so each one can be considered as a set of transformed PII fields. In addition, there are overlapping PII fields populated within different hash codes. Therefore, set theory may be used to systematically validate questionable PII fields. As long as a hash code is matched, its corresponding PII fields may be eliminated from questionable PII fields by set operations. Because missing values of optional PII fields are permitted, first all probable combination patterns of PII fields for perfect or good hash codes need to be analyzed and then the algorithm for checking questionable PII input might be designed.

### Probable PII Combination Patterns for Perfect or Good Hash Codes

**Figure 2:**
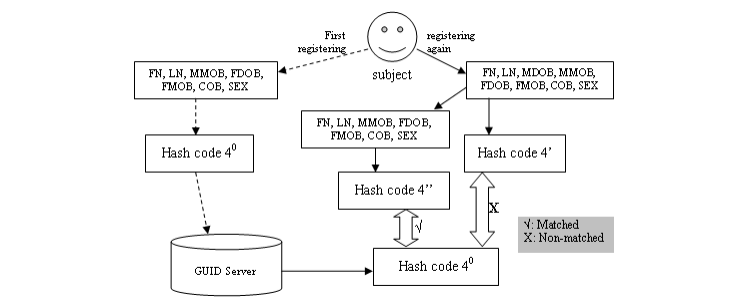
An example for match among hash codes.

According to the principle of the GUID system, there are 3 types of hash codes and a subject is identified only with perfect or good hash codes. Missing fields may affect the match of a hash code. While registering a subject, if missing fields are considered, some improper mismatching will be avoided. For example, hash code 4 from Table 2 (Figure 2) is generated from the combination of required fields FN, LN, COB, and SEX and optional fields MDOB, MMOB, FDOB, and FMOB. Assuming that a subject was registered for the first time, the MDOB field was missed, and the other fields were correctly inputted, it would generate hash code 4^0^. But when the subject is registered again on another site, and the correct value of all the above PII fields including MDOB is provided, the system will produce hash code 4’. Because field MDOB was missed in hash code 4^0^, hash code 4’ will not match with hash code 4^0^. However, there is a perfect match between hash code 4’ and hash code 4. If field MDOB is supposed as missing field to generate hash code 4’’, hash code 4’’ will be a perfect match with the previous hash code 4^0^ and thus will avoid improper mismatching of hash code 4. So all perfect or good hash codes of a subject, which are registered, should be analyzed for identifying the subject and checking questionable PII fields.

**Figure 3:**
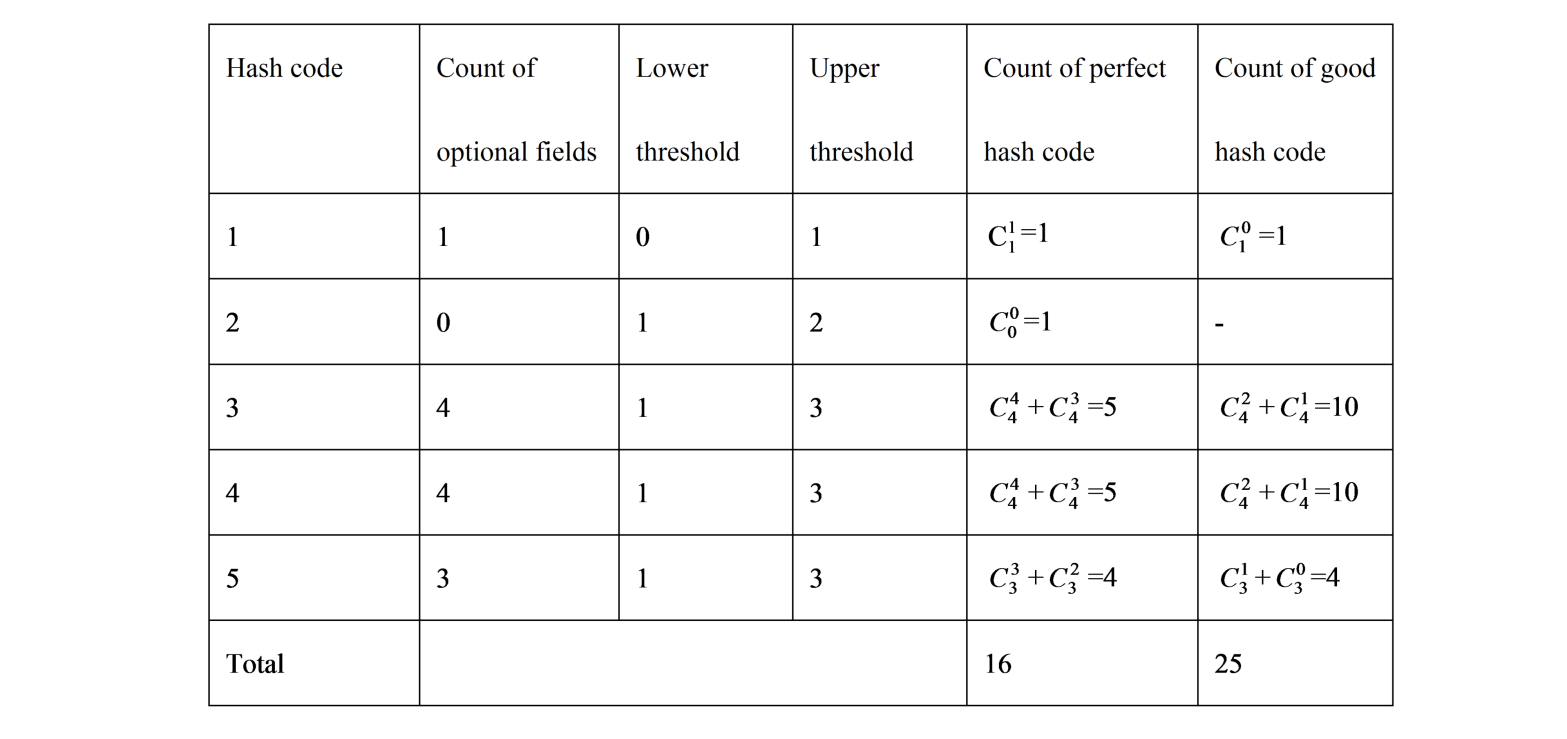
The count of probable perfect or good hash codes.

Each hash code is generated from different combination patterns of PII fields, which are optional or required. Based on the combination patterns, the match rule of hash code and the type of PII fields, all probable perfect or good hash codes of the GUID system can be analyzed and identified (Figure 3 and Table 4). For example, hash code 3 is generated from a combination pattern of fields MFN, MLN, FFN, FLN, FN, and YOB. Of them, fields FN and YOB are required fields and the other 4 fields are optional. According to match rules of hash codes, a perfect hash code 3 may have 1 missing field and a good hash code 3 may have 2 or 3 missing fields. That is, a perfect hash code 3 may contain 1 missing field from MFN, MLN, FFN, or FLN and a good hash code 3 may use only 1 or 2 of those PII fields. So there are 5 probable perfect and 10 probable good hash code 3.

**Table 4:** Probable personally identifiable information (PII) combinations for hash codes with different matching types.

Index	Hash code	Combinations of personally identifiable information fields								Missed fields	Type of hash code
1	1	GIID	SEX	DOB	YOB						Perfect
2		a	SEX	DOB	YOB					GIID	Good
3	2	FN	LN	MN	DOB	MOB	COB				Perfect
4	3	MFN	MLN	FFN	FLN	FN	YOB				Perfect
5		MLN	a	FFN	FLN	FN	YOB			MFN	Perfect
6		MFN	FFN	a	FLN	FN	YOB			MLN	Perfect
7		MFN	MLN	FLN	a	FN	YOB			FFN	Perfect
8		MFN	MLN	FFN	FN	a	YOB			FLN	Perfect
9		a	a	FFN	FLN	FN	YOB			MFN, MLN	Good
10		a	MLN	a	FLN	FN	YOB			MFN, FFN	Good
11		a	MLN	FFN	a	FN	YOB			MFN, FLN	Good
12		MFN	a	a	FLN	FN	YOB			MLN, FFN	Good
13		MFN	a	FFN	a	FN	YOB			MLN, FLN	Good
14		MFN	MLN	a	a	FN	YOB			FFN, FLN	Good
15		a	a	a	FLN	FN	YOB			MFN, MLN, FFN	Good
16		MFN	a	a	a	FN	YOB			MLN, FFN, FLN	Good
17		a	MLN	a	a	FN	YOB			MFN, FFN, FLN	Good
18		a	a	FFN	a	FN	YOB			MFN, MLN, FLN	Good
20	4	MDOB	MMOB	FDOB	FMOB	FN	LN	SEX	COB		Perfect
19		a	MMOB	FDOB	FMOB	FN	LN	SEX	COB	MDOB	Perfect
21		MDOB	a	FDOB	FMOB	FN	LN	SEX	COB	MMOB	Perfect
22		MDOB	MMOB	a	FMOB	FN	LN	SEX	COB	FDOB	Perfect
23		MDOB	MMOB	FDOB	a	FN	LN	SEX	COB FMOD	Perfect	
24		a	a	FDOB	FMOB	FN	LN	SEX	COB	MDOB, MMOB	Good
25		a	MMOB	a	FMOB	FN	LN	SEX	COB	MDOB, FDOB	Good
26		a	MMOB	FDOB	a	FN	LN	SEX	COB	MDOB, FMOB	Good
27		MDOB	a	a	FMOB	FN	LN	SEX	COB	MMOB, FDOB	Good
28		MDOB	a	FDOB	a	FN	LN	SEX	COB	MMOB, FMOB	Good
29		MDOB	MMOB	a	a	FN	LN	SEX	COB	FDOB, FMOB	Good
30		a	a	a	FMOB	FN	LN	SEX	COB	MDOB, MMOB, FDOB	Good
31		a	a	FDOB	a	FN	LN	SEX	COB	MDOB, MMOB, FMOB	Good
32		a	MMOB	a	a	FN	LN	SEX	COB	MDOB, FDOB, FMOB	Good
33		MDOB	a	a	a	FN	LN	SEX	COB	MMOB, FDOB, FMOB	Good
34	5	FN	MN	MFN	FFN	MLN	MOB				Perfect
35		FN	MN	a	FFN	MLN	MOB			MFN	Perfect
36		FN	MN	MFN	a	MLN	MOB			FFN	Perfect
37		FN	MN	MFN	FFN	a	MOB			MLN	Perfect
38		FN	MN	MFN	a	a	MOB			FFN, MLN	Good
39		FN	MN	a	a	MLN	MOB			MFN, FFN	Good
40		FN	MN	a	FFN	a	MOB			MFN, MLN	Good
41		FN	MN	a	a	a	MOB			MFN, FFN, MLN	Good

^a^The optional field that may be missed while being collected.

### Set Theory and Checking Questionable Fields

Set theory is one of the most important theories of information processing. A set is a collection of a type of objects, and its basic operations include subtraction, union, intersection, subset, and so on. To eliminate some elements from a collection, the set operation (ie, subtraction) is a good solution. Since a hash code is transformed from a combination of PII fields, it must be related to a set of PII fields. Once it matches with one of the hash codes of an identified subject, a corresponding set of PII fields also must match with each other and those PII fields will be considered validated. So using set theory, with the match rule of hash codes and subject in the GUID system, some PII input errors are likely to be located. For example, assuming that while registering a subject, it is found that the PII fields for hash codes 3, 4, and 5 are without missing fields and those hash codes match perfectly with the corresponding hash codes of the identified subject in the server. In addition, hash codes 1 and 2 do not match with the corresponding hash codes of the identified subject. According to the matching rules of the subject, it may be deduced that the subject has been registered in the system. The PII fields related to hash codes 3, 4, and 5 can be eliminated from questionable PII fields. That is,

{GIID, FN, LN, MN, DOB, MOB, YOB, SEX, COB, MFN, MLN, FFN, FLN, MDOB, MMOB, FDOB, FMOB}

/{FN,YOB, MFN, MLN, FFN, FLN} //PII related to hash code 3

U {FN, LN, MDOB, MMOB, FDOB, FMOB, COB, SEX} //PII related to hash code 4

U {FN, MN, MOB, MFN, FFN, MLN} //PII related to hash code 5

={GIID, DOB}

**Figure 4:**
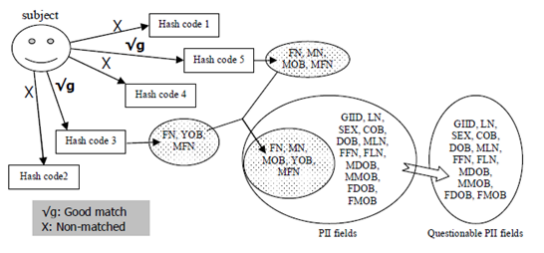
An example for locating questionable personally identifiable information (PII) fields while hash codes are perfect match.

**Figure 5:**
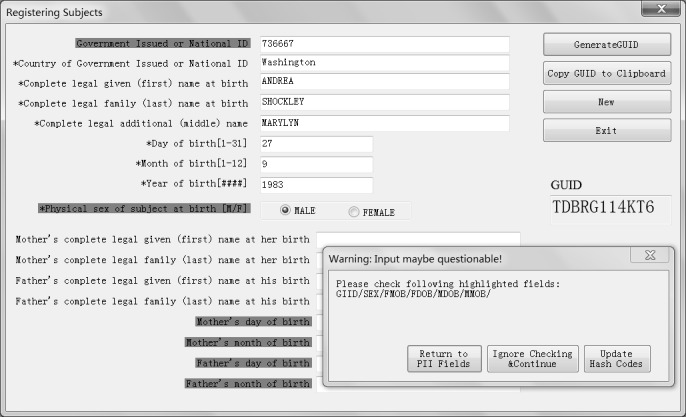
An example for locating questionable personally identifiable information (PII) fields while hash codes are good match.

Therefore, the result suggests that questionable fields may be located at PII fields DOB and GIID (Figure 4). Figure 5 shows another example of locating questionable PII fields. In this case, only hash code 3 and 5 are good matches and PII fields FFN, FLN, and MLN are missed. There are 2 good hash code matches (G=2), so the subject has been registered according to the match rules. Due to the missing of fields FFN, FLN, and MLN, here the PII fields corresponding to hash code 3 are FN, YOB, and MFN and those to hash code 5 are FN, MN, MOB, and MFN. Therefore,

{GIID, FN, LN, MN, DOB, MOB, YOB, SEX, COB, MFN, MLN, FFN, FLN, MDOB, MMOB, FDOB, FMOB}

{ FN, YOB, MFN } U {FN, MN, MOB, MFN } //PII related to hash code 3,5

={ GIID, LN, SEX, COB, DOB, MLN, FFN, FLN, MDOB, MMOB, FDOB, FMOB}

It may be deduced that data entry error exists within PII fields GIID, LN, SEX, COB, DOB, MLN, FFN, FLN, MDOB, MMOB, FDOB, and FMOB.

Based on set theory and the principle of the GUID system, while registering subjects, the algorithm checking questionable PII fields can be described as following.

Step 1 Input PII of subject *S*_r_ being registered;

Step 2 Generate all probable perfect or good hash codes *HC*_pg_ of *S*_r_, *HC*_pg_={HC_1_, HC_2_, …, HC_41_}, and store temporarily their corresponding set of PII field name, *PII*_1_*, PII*_2_*, ..., PII*_41_*,* to *HC*_1_*, HC*_2_*, ...* and *HC*_41_ on the local site as described in Table 4:

*PII*_1_={GIID, SEX, DOB, YOB}

*PII*_2_={SEX, DOB, YOB}

…

*PII*_41_={ FN,MN,MOB}

Step 3 Find matched subjects, *S*_m_, with *S*_r_ from the GUID server according to match rules and *HC*_pg_;

Step 4 If count of *S*_m_*>1* then

*S*_r_ is not unique;

else if *S*_m_ is empty then

*S*_r_ is a new subject;

else

Find hash codes in *HC*_pg_ that match with those of *S*_m_

and get their set of PII fields, *PII*_1_^’^*, PII*_2_^’^*, ...*;

Step 5 Calculate union *U*_PII_ of *PII*_1_^’^*, PII*_2_^’^*, …*;

*U*_PII_*= PII*_1_^’^U *PII*_2_^’^*,* U *…*

Step 6 Calculate subtraction between *U*_PII_ and all PII fields;

*R*_PII_ = {GIID, FN, LN, MN, DOB, MOB, YOB, SEX, COB, MFN, MLN, FFN, FLN, MDOB, MMOB, FDOB, FMOB} - *U*_PII_

Step 7 Return remaining PII fields *R*_PII_ which are questionable.

### Simulations

For evaluating the proposed algorithm, the mailing list information [18] has been used as simulation data. Of mailing list information on 1 million individuals, first name (FN), last name (LN), and middle name (MN) were kept and the city of residence was used as city of birth (COB). Dates of birth (YOB, MOB, and DOB) were randomly generated. Individuals were assigned parents’ information (MFN, MLN, FFN, FLN, MDOB, MMOB, FDOB, and FMOB) to be logically consistent with the family structure. The values of field GIID are replaced with the index of subjects. Randomly emptying is used to simulate missing of optional fields. From the included pretreated subjects, we randomly selected 200,000 subjects for the simulation study of our method. Their original hash codes were generated and stored on the GUID server.

Then we randomly planted 200,000 errors into the simulation data, including emptying, inserting, deleting, and replacing. In any given field of the same hash code, the count of planted error is not more than one. After planting errors, out of 200,000 subjects, there are 127,700 subjects with errors and 72,300 subjects with no error. In 1 subject, the maximum for planted errors is 8. The count (N_Err) and percent of planted errors by PII fields is shown in Table 5.

**Table 5:** Distribution of planted errors by personally identifiable information (PII) fields.

PII^a^ fields		N_Err	Percent (%)
**Required fields**			
	FN	12,937	6.47
	LN	14,166	7.08
	MN	10,234	5.12
	COB	12,954	6.48
	DOB	10,440	5.22
	MOB	12,645	6.32
	YOB	11,578	5.79
	SEX	11,587	5.79
**Optional fields**			
	GIID	7980	3.99
	MFN	12,984	6.49
	MLN	10,504	5.25
	FFN	10,823	5.41
	FLN	11,656	5.83
	MDOB	13,603	6.80
	MMOB	11,301	5.65
	FDOB	11,188	5.59
	FMOB	13,420	6.71
Total		200,000	100

^a^PII: personally identifiable information.

After the dataset is treated, only error-planted subjects are used for simulating input while registering from the client application. The proposed algorithm is applied to validate and locate these planted errors.

### Applications

When reregistering a subject in a GUID system, the proposed methods may be used to perform the following 2 tasks:

1. Checking questionable PII fields to ensure correct input. If any of the PII fields of the subject are improperly input, the client application will prompt the user to recheck the specified PII without revealing actual input value by using the proposed method.

2. Updating hash codes. If the client ensures that input of PII fields are correct and more complete than before, the application will allow the system to update hash codes.

For the above 2 tasks, we have developed an application program and integrated it into current GUID registering operation. Registered subjects are selected to confirm its value.

## Results

### Matching of Subjects

Due to planted errors, the values of some PII fields have changed. As shown in Table 6, of 127,700 error-planted subjects, 89.63%(114,464/127,700) are still identified by the hash codes from their remaining correct PII fields. The other 10.37%(13,236/127,700) subjects cannot match with their previous entries and are identified as new subjects. 83.16% (65,383/78,619) of the subjects with errors in required fields are still identified. All unidentified subjects have the required PII fields with errors. Additionally, of all identified subjects, 57.13% (65,383/114,464) have the required fields with errors.

**Table 6:** Identifying of error-planted subjects.

Matching type	Rec_erf_^a^	Rec_nerf_^b^	Subtotal
Unidentified	13,236	0	13,236
Identified	65,383	49,081	114,464
Total	78,619	49,081	127,700

^a^Rec_erf_: the count of subjects with errors in required fields.^b^Rec_nerf_: the count of subjects with no error in required fields.

Simulation results show that the average errors planted into the identified subjects is 1.48 and that planted into the unidentified subjects is 2.29. Table 7 lists the count of errors planted into 1 subject (n_Err_), the count of subjects with n_Err_ errors (n_Rec_Err_), the count of identified subjects with n_Err_ error, and the ratio of n_Rec_Err_Mtch_ to n_Rec_Err_ (n_Rec_Err_Mtch_). Table 8 displays the count of incorrect required fields in 1 subject (n_Err_ReqF_), the count of subjects with n_Err_ReqF_ incorrect required fields (n_Rec_Err_ReqF_), the count of identified subjects with n_Err_ReqF_ incorrect required fields (n_Rec_Err_ReqF_Mtch_), and the ratio of n_Rec_Err_ReqF_Mtch_ to n_Rec_Err_ReqF_.

**Table 7:** Identifying of subjects with different count of planted errors.

n_Err_	n_Rec_Err_	n_Rec_Err_Mtch_	Ratio
1	74,883	71,796	95.88
2	37,327	32,104	86.01
3	12,143	8798	72.45
4	2792	1545	55.34
5	476	199	41.81
6	69	18	26.09
7	8	4	50.00
8	2	0	0.00

**Table 8:** Identifying of subjects with different count of error required fields.

n_Err_ReqF_	n_Rec_Err_ReqF_	n_Rec_Err_ReqF_Mtch_	Ratio
0	49,081	49,081	100.00
1	62,716	56,750	90.49
2	14,026	8038	57.31
3	1740	569	32.70
4	132	25	18.94
5	5	1	20.00

### Recalling of Planted Errors

Simulation results show that PII errors may be found and located within the limited fields. The best situation is to precisely locate an error at 1 PII field. The worst situation is to reduce the questionable scope of errors down to a set of 13 PII fields. According to the simulated results, the mean questionable scope of errors is shrunk to a set of 5.64 PII fields, 3.59 times as many as the average of errors planted into a subject. It suggests that the mean questionable scope of errors can be limited to a set of less than 4 PII fields.

For identified subjects, the count of analyzed questionable PII fields (n_cqf_) is related to the count of planted errors in a subject (Table 9). For example, for subjects with only 1 error, the average of questionable PII is shrunk to 4.27 fields. For those with 7 errors, it is limited to 13 fields.

Table 10 lists the count of analyzed questionable fields by PII fields (n_cqf_PII_). The subjects with error field FN has the maximum mean analyzed questionable PII (13 fields) and the subjects with error field GIID has the minimum mean analyzed questionable PII (3.74 fields). The subjects with other error PII fields have no significant difference.

If only 1 error is planted into a subject, the count of analyzed questionable PII fields (n_cqf_1_) depends on the type of error PII field (Table 11). For example, it is 1 for the error field GIID, 13 for the error field FN, and 1 or 4 for the error field MDOB.

**Table 9:** The count of analyzed questionable fields by count of errors.

Count of planted errors in a subject	n_cqf_		
	Minimum	Maximum	Average
1	1	13	4.27
2	2	13	7.39
3	3	13	9.42
4	4	13	10.86
5	6	13	11.67
6	11	13	11.83
7	13	13	13.00

**Table 10:** The count of analyzed questionable fields by personally identifiable information (PII) fields.

PII^a^ fields with planted errors		n_cqf_PII_		
		Minimum	Maximum	Mean
**Required fields**				
	FN	13	13	13
	LN	6	13	7.65
	MN	2	13	5.56
	SEX	6	12	7.30
	COB	6	13	7.67
	DOB	2	11	5.69
	MOB	2	13	5.53
	YOB	3	11	5.28
**Not required fields**				
	GIID	1	11	3.74
	MFN	1	13	6.48
	MLN	1	13	6.51
	FFN	1	13	6.59
	FLN	1	13	4.84
	MDOB	1	13	6.12
	MMOB	1	13	6.11
	FDOB	1	13	6.09
	FMOB	1	13	6.06

^a^PII: personally identifiable information.

**Table 11:** The count of analyzed questionable personally identifiable information (PII) fields from subjects with only one error.

PII^a^ fields with planted errors		n_cqf___1_
**Required fields**		
	FN	13
	LN	6
	MN	2
	SEX	6
	COB	6
	DOB	2
	MOB	2
	YOB	3
**Optional fields**		
	GIID	1
	MFN	1/4
	MLN	1/4
	FFN	1/4
	FLN	1
	MDOB	1/4
	MMOB	1/4
	FDOB	1/4
	FMOB	1/4

^a^PII: personally identifiable information.

### Applications

**Figure 6:**
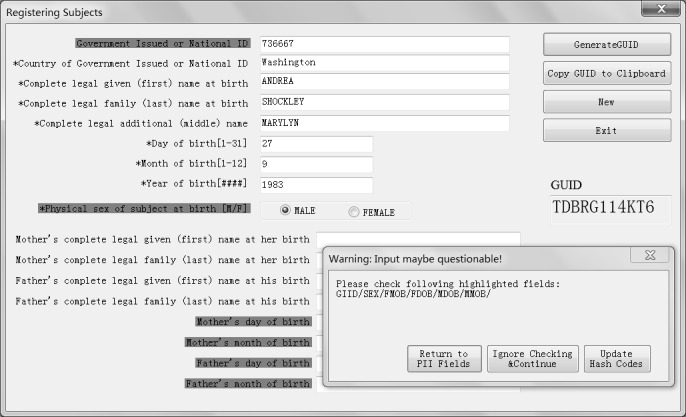
The application of checking questionable personally identifiable information (PII) fields.

The proposed hash code analysis scheme is integrated into the GUID application to enhance GUID accuracy. While registering a subject, who has been previously registered in the system, it analyzes the questionable PII fields, highlights them, and requests the client to correct them (Figure 6).

When the application finds the questionable PII fields, it will give a hint regarding possible PII errors. If it is confirmed that the input of all PII fields are proper, the user may select “update hash codes” function and the application will update the hash codes in the server based on user’s input.

## Discussion

### Identifying of Subject

In the GUID system [18], there are 17 PII fields, including 8 required fields and 9 optional fields. PII fields are combined into 5 patterns, which are processed into hash codes by a one-way hash algorithm. For privacy protection, only hash codes and its related random GUID code are stored on the server. In this case, it is impossible to directly identify a subject by PII and hash codes are the key to identifying a subject. One perfect hash code or 2 good hash codes is sufficient to identify a subject and the system has better error tolerance. A subject with error PII fields may still be identified and it is confirmed by the simulation result of this study. As shown in Table 6, 89.63% of subjects with error PII fields do still match with their previous entries.

In addition, simulation results also show that the count and type of error PII fields in a subject have great effect on identifying the subject. In Table 7, it can be found that the probability of identifying the subject is reversely related to the count of planted errors. That is, the more errors that are planted into a subject, the lower is probability of identifying the subject. Table 6 shows that all unidentified subjects have the errors within its required PII fields. It can also be deduced that the subject without error within required PII fields must be correctly identified. That is, if all required PII fields of a subject are correctly entered, the subject must be identified well. Table 8 indicates that when more errors are planted into required PII fields of a subject, the probability of identifying the subject is lower. Therefore, it suggests that required PII fields are vital to identifying a specific subject. According to the principles of the GUID system, we can also find that the match criteria and find important PII fields based on the composition of hash codes. For example, PII field FN is a required field for hash code 2, 3, 4, and 5. Once this PII field of a subject is incorrect, those 4 hash codes will not be matched. In turn, it will significantly reduce the probability of identifying the subject. So to ensure correct registration of a subject, especially with required PII fields, correct data entry is critical to avoiding false splits.

### Reducing PII Entry Errors

Hash codes are generated from PII, but it is an irreversible process and a hash code cannot be transformed back into PII. Therefore, it is impossible to validate questionable input by reversing hash codes to PII, which is intended by design. Additionally, missing values of PII fields make it more difficult to validate questionable PII fields. Fortunately, there exists a map between combinations of PII fields and hash codes and there are overlapping PII fields among hash codes of a subject. Each hash code represents a set of PII fields and all probable perfect or good hash codes (Figure 3 and Table 4) may be analyzed and produced for a subject being registered. Therefore, set theory can be used for analyzing questionable PII fields. For example, while registering a subject, if its hash code 1 is perfectly matched, then its PII fields GIID, SEX, DOB, and YOB can be eliminated from questionable PII fields. Simulation results confirm that the questionable PII fields of all identified subjects may be found and located. The best situation is to locate an error at one exact PII field; the worst situation is to reduce the scope of possible errors in a subject down to a set of 13 PII fields. The mean scope of possible errors in a subject is shrunk to a set of 5.64 PII fields, 3.59 times as many as the average of errors planted into a subject.

The simulation results also show that the count of analyzed questionable PII fields is closely related to the count of actual errors. The greater the count of actual errors, the more the questionable PII fields to be evaluated (Table 9). For subjects with only 1 error, the scope of questionable inputs can be limited to an average set of 4.27 PII fields. For subjects with 7 errors, it could be a set of 13 PII fields. The type of PII fields with error is also associated with the count of analyzed questionable PII fields. For subjects with only 1 error, if the error is for an optional PII field, it can be located at 1 or upto 4 PII fields. If the error is for a required field, it cannot be limited to such narrow scope (Table 11). For example, the error in the FN field will result in the failed matching of hash codes 2, 3, 4, and 5 no matter whether there are other errors. Thus, at most, only hash code 1 is a perfect match and fields GIID, SEX, DOB, and YOB can be eliminated from questionable fields. The remaining 13 PII fields will be evaluated as questionable fields (Tables 10 and 11). Fortunately, the accuracy of first name is very high [18].

By using the proposed method in this study, while registering a subject, the application may give a proper hint to the user about questionable PII input. If the user assures that input of PII fields are correct, the hash codes in the system may be updated to improve from the previous entry error, thus improving the robustness of the GUID system.

### Conclusions

In summary, a subject with PII errors may still be identified in the GUID system but it depends on the number and type of PII errors. Using set operations, questionable PII fields from the client application may be analyzed based on hash codes but it is difficult to find the exact location of an error because hash codes come from combinations of PII fields and it cannot be reversed to PII. If questionable PII fields need be precisely located, all probable perfect or good hash codes must be stored on the server or the generating mechanism of hash codes in the system must be redesigned.

## Abbreviations

CNOB: Country of birth
COB: Name of city or municipality in which subject was born
DOB: Day of birth
FDOB: Father’s day of birth
FFN: Father’s complete legal given (first) name at his birth
FLN: Father’s complete legal family (last) name at his birth
FMOB: Father’s month of birth
FN: Complete legal given (first) name at birth
GIID: Government Issued or national ID
GUID: Global Unique Identifier
LN: Complete legal family (last) name at birth
MDOB: Mother’s day of birth
MFN: Mother’s complete legal given (first) name at her birth
MLN: Mother’s complete legal family (last) name at her birth
MMOB: Mother’s month of birth
MN: Complete legal additional (middle) name
MOB: Month of birth
PII: Personally identifiable information
SEX: Physical sex at birth (male or female)
YOB: Year of Birth
